# Drug importation: limitations of current proposals and opportunities for improvement

**DOI:** 10.1038/s41408-021-00522-3

**Published:** 2021-07-15

**Authors:** Caleb J. Scheckel, S. Vincent Rajkumar

**Affiliations:** Division of Hematology, Department of Medicine, Mayo Clinic, Rochester, MN USA

**Keywords:** Public health, Therapeutics

## Abstract

Drug importation is a policy proposal to help alleviate rising pharmaceutical prices. Restrictions on drug importation stem from the Federal Food, Drug, and Cosmetic Act but authorization of importation can be made by the Health and Human Services (HHS) Secretary. During the Trump administration a number of states passed laws to develop a drug importation programs, however, none have been authorized by HHS. Limitations of these importation programs include sole reliance on Canada, exclusion of high-cost drugs like biologics, and persistent legal hazard of the Personal Importation Program. Potential revisions to current law include expansion of countries approved for importation, inclusion of biologics, and codifying protection for personal importation. Drug importation policies are not a panacea to address rising pharmaceutical prices but may blunt prices while more permanent solutions are pursued.

## Introduction

As part of the larger discourse on rising healthcare expenditures in the U.S., the affordability of prescription drugs continues to occupy a significant position in health policy debate with the majority of Americans believing that lowering their cost is a top health care priority. [1, 2] Prescription drugs account for 10% of overall health spending in the U.S. with prescription sales in 2016 exceeding $448 billion. [3]

In cancer, where pharmaceutical spending represents upwards of 20% of total spending, the issue is especially salient. Considerable therapeutic advances in the treatment of hematologic neoplasms have resulted in marked improvements in overall survival and resulted in some of these conditions being treated like chronic diseases. [4, 5] However, many of these agents are novel and not yet subject to generic or biosimilar price pressure. Patients are potentially thus confronted with high out-of-pocket expenses for years on end.

A study by the Kaiser Family Foundation showed median annual out-of-pocket expenses of Medicare beneficiaries in 2019 for the following common drugs used in various hematologic malignancies: venetoclax $8,712, imatinib $8,983, acalabrutinib $10,175, midostaurin $11,830, and lenalidomide $14,461. The correlate median annual total costs were: $135,686, $91,844, $171,481, $206,243, and $259,051, respectively. [6] The financial burden of these therapies has yielded renewed interest in alternative methods to reign in cost. Consumers and policymakers alike have highlighted the significant differences in cost between the U.S. and other industrial nations. [7] In this context, much attention has been devoted to large-scale importation at the state or national level.

## Background and recent discussion

‘Drug importation’ is the practice of importing prescription drugs that were manufactured, either domestically or abroad, with the intent for sale in another country. Importation (used interchangeably with re-importation by many policymakers) typically occurs on an individual level when Americans import drugs for personal use by either filling their prescriptions in Canada or Mexico or through mail-order/internet pharmacies. The Food and Drug Administration (FDA), under the Federal Food, Drug, and Cosmetic Act (FDCA) of 2003, regulates prescription drugs in the U.S. Current law prohibits the importation of a U.S.-manufactured drug by anyone other than the manufacturer (FDCA Section 801(d)(1)(A)). [8] Individuals who import pharmaceuticals for personal use do so under the FDCA’s personal importation policy (PIP) which allows the FDA discretion in enforcing the importation prohibition. While technically approved on a case-by-case basis, lenience is often given to individuals who import a small quantity (e.g., a 90 day supply) if the product is not deemed to pose an unreasonable risk, and individuals certify in writing that the product is for personal use only. [9]

Options for larger scale importation are prohibited with several notable exceptions. Under FDCA Section 804, the Health and Human Services (HHS) Secretary can authorize a drug importation program whereby pharmacists and wholesalers could import unapproved prescription drugs from Canada into the U.S. under controlled circumstances. Such a program would be required to demonstrate no additional risk to the public’s health and safety and would have to offer “significant reduction in the cost” to U.S. consumers. Other exceptions to drug importation revolve around addressing emergency medical needs or extreme cases of supply chain disruption. Such programs would vary with US administrations similar to federal prosecution for marijuana possession and use. Interestingly, the FDCA definition of prescription drugs under Section 804 excludes controlled substances, biologic products, and medications that are either infused or injected intravenously. These are notable exclusions because, for example, while biologics represent only 2% of all U.S. prescriptions, in 2017 they accounted for 37% of drug spending. [10]

The Trump administration renewed public interest in drug importation as a potential strategy for curbing rising pharmaceutical prices, and, in September 2020, the HHS released its final rule on “Importation of Prescription Drugs”. [11] The plan calls for the development of Section 804 Importation Programs (SIPs) by which states, tribes, wholesalers, or pharmacists could import Canadian drugs into the U.S. Eligible drugs would be restricted to those approved by Health Canada. This final rule is being challenged by the Pharmaceutical Research and Manufacturers of America, an industry trade group, with a suit filed November 2020. [12]

Under this flurry of recent interest, numerous states have either enacted drug importation laws (Colorado, Florida, Maine, New Hampshire, New Mexico, and Vermont), have pending legislation, or exploratory committees on the topic. Notably there are several states where drug re-importation legislation has failed (North Dakota, Oregon, Utah, West Virginia, and Wyoming). [9] After state law is enacted, the proposed re-importation program must then be certified by the HHS secretary before becoming active. Reflecting the multiplicity of authors and state-specific interests, these importation program proposals are heterogeneous, but model legislation does exist. [9, 13] As of 1 March 2021, it remains to be seen whether the Biden administration will defend the importation policy issued under the prior administration.

## Stakeholder arguments

The American Medical Association (AMA) and the Association of American Retired Persons (AARP), along with other organizations, recognize the strong public sentiment on the topic of drug affordability and have expressed support for policies that would provide for importation of lower-cost drugs for personal use in a way that ensures drug safety and integrity. [14, 15] These organizations recognize that the FDA lacks the power to oversee another country’s distribution systems, and therefore cannot fully guard against the re-importation of counterfeit, expired, contaminated, or drugs stored under unsafe conditions. Recent examples of counterfeit therapies distributed through misleading online pharmacies exist. [16] However, these risks must be contrasted with the risks associated with consumers not taking prescribed medicines simply because they are unaffordable. Notably, the FDA has expressed confidence in Health Canada, the Canadian agency tasked with ensuring the integrity of pharmaceutical manufacturing, importation, and distribution, with regards to providing effective oversight for drugs approved for Canadian patients. [17]

Governments recognize the potential savings for their citizens associated with drug importation, and this has spurred the recent activity of state-sponsored drug importation policy proposals. In a 2018 report and analysis on Vermont’s proposed wholesale importation program, the differences between Canadian and major insurance carrier pharmaceutical costs based on just 17 high-spend drugs would yield savings of $1–5 million annually. [18]

The pharmaceutical industry opposes the practice of importation primarily because such programs would result in lapses in patient safety and the potential disruption to the recovery of investment in research and development (R&D) costs. They argue that the significant investment of time and money, not to mention regulatory hurdles, that is required to take pharmaceutical concepts from bench to bedside justifies the end cost to U.S. consumers and that the loss of profits from drug importation would ultimately stifle innovation. [19] However, there is suggestive research that the drug premium charged to U.S. consumers far exceeds the R&D spending by the pharmaceutical industry. [20]

Lastly, drug importation, as currently permitted in the FDCA, would rely solely on products from Canada. From a Canadian perspective, depending on the scope of adoption in the U.S., this could lead to product shortages for Canadian consumers thus sacrificing Canadian accessibility to therapies for the sake of American affordability. Whether shortages would reflect market forces, intentional restriction on manufacturing at the behest of the pharmaceutical industry, or both, is a matter of debate but historical examples of each can be seen. [21, 22]

## Limitations of suggested changes

State governments are increasingly turning to drug importation as a means to lower the cost of drugs for their citizens. Under the purview of the Trump administration, 6 states have signed drug importation proposals into law and there is pending legislation in many others. Consistent with the language in the FDCA, all such programs would rely solely on slack in the Canadian pharmaceutical market to achieve their cost savings. With a population of only 37.6 million [23], it seems unlikely that the Canadian pharmaceutical marketplace could realistically or sustainably absorb U.S. drug demand, especially as additional state importation programs are signed into law. Furthermore, many of the therapies excluded from importation under the FDCA are significant drivers of the rise in pharmaceutical spending. The feasibility of constructing a state-run program that could ensure authenticity, purity, potency, and lack of adulteration congruent with FDA standards while still achieving consumer savings is yet another issue. Lastly, the enforcement ambiguity of the PIP currently exposes participants to the stigma of running afoul of federal law and could reasonably undermine broad participation. To that end, we propose several revisions to Section 804 of the FDCA and its downstream effects on FDA policy that could address the above concerns.

## Proposals for improvement

The first revision would be expanding the list of approved countries that the U.S. could import from to include: Australia, Canada, Israel, Japan, New Zealand, Switzerland, South Africa, a member-state of the European Union, or a country in the European Economic Area. This is consistent with the countries listed in HR 934, the Personal Drug Importation Fairness Act of 2017 that was introduced by Congressman Keith Ellison (D-MN). [24] Federal regulation already ensures the safety of foreign manufactured products that ultimately come to the U.S. for consumption. These same FDA-registered manufacturers supply pharmaceuticals to countries that would be open to importation under the proposed rules. Furthermore, U.S. standards for manufacturing and handling of pharmaceuticals are comparable to those of many other countries. Because of the congruency of practice, the European Union and the FDA accept each other’s inspection findings for manufacturing plants within their borders. Similar reciprocity is seen between the U.S. and Canada. By expanding the catchment from multiple industrial nations, U.S. consumers would enjoy similar safety requirements as imposed by the FDA while being able to tap into a larger supply of drugs.

The second revision would include redefining what constitutes a prescription drug to include biologics and therapies that are either infused or injected intravenously. This includes prescriptions filled under a Risk Evaluation and Mitigation Strategy (REMS) program as long as the prescriber follows the same rules of patient registration as they would normally. According to the 2017 report from IQVIA, they note that the largest proportion of new medicines launched in the last five years has been specialty drugs and these therapies now account for 46.5% of pharmaceutical spending. [7] This trend is expected to grow. However, many of these therapies are ineligible for importation under present US laws and regulations. While the current FDCA definition includes therapies that the bulk of consumers may actually need because the medical conditions which they treat are common (hypertension, hyperlipidemia, and depression), it excludes therapies that often carry the largest price tag and inherently the greatest risk of yielding financial toxicity.

A third revision would seek to simplify supply chain monitoring and the process by which drug fidelity is assured. The FDCA mandates that an importation program must “impose no additional risk” to consumer safety while yielding savings. Section 804 requires “that safeguards be in place to ensure that each prescription drug imported under the regulations complies with [the New Drug Approval requirements under] section 505” and that the importer or manufacturer of the drug “meets all labeling requirements under this Act.” These provisions are formidable obstacles to achieving “significant cost savings” required under the statute. While proposals for cost-effective track and trace compliance under the Drug Supply Chain Security Act (DSCSA) exist [25], it remains unclear whether a state or other entity could reasonably assume the responsibilities of the FDA for such a program. In the absence of the economies of scale enjoyed by the FDA, states would likely find that a significant investment in personnel would be necessary to ensure drug safety. How this impacts overall savings is unclear. It is conceivable that consumers could still enjoy some cost savings but at the expense of a larger state government. Whether this transfer of cost is appetizing to voters would be state-dependent. Though an easy solution for reconciling safety and cost does not exist, we envision a system where (1) pharmaceuticals manufactured at FDA-approved locations are (2) labeled and tracked to an FDA-approved distributor or retail organization in a foreign market, and (3) tracked to U.S. and finally end consumers using existing pharmaceutical shipping and transportation infrastructure.

The final revision would include codifying language surrounding the FDA’s PIP. Under current law, personal importation is technically illegal with consumers assuming the risk of pharmaceutical confiscation, penalization, and even prosecution. Though the FDCA details that the “Secretary should—(A) focus enforcement on cases in which the importation by an individual poses a significant threat to public health; and (B) exercise discretion to permit individuals to make such importations,” the degree of determent that the current system causes interested but risk-averse consumers is unknown. Proposed amendment to the FDCA’s PIP language would include legalization of personal importation as long as consumers have a: (1) valid prescription from a licensed healthcare provider, (2) for a supply no >90 days, (3) that is intended for personal use only, (4) and that the user assumes the risk of obtaining a pharmaceutical outside of the normal FDA-approved supply chain regulation. While personal importation is not geographically or financially feasible for many consumers, constructing a list of verified and reputable online pharmacies with physical presence abroad may expand access to a larger segment of the U.S. population.

## Lingering questions

Although the proposed changes would offer drug importation programs an improved chance of positively impacting market prices on pharmaceuticals for consumers, several legitimate questions remain. One major concern surrounds the ultimate impact of importation on prices paid by insurance networks. For example, in its current construct, a complex drug rebate system determines the final drug price paid by Medicaid programs. Due to complexities in the structure of the rebate calculation, there is concern that lower drug list prices from importation and its negative impact on inflationary rebates could paradoxically increase net costs to the Medicaid system. Stemming in part from this concern, language in HHS’s 2019 “Safe Importation Action Plan” excludes participation of Medicaid programs in drug importation. [26] Alas, the government cannot require a manufacturer to pay a rebate on a drug it did not sell to Medicaid in the first place.

Another concern is the out-of-pocket (OOP) costs assumed by the insured. If insured patients choose to buy imported drugs because of lower OOP costs during their deductible period, they essentially double-pay for drugs because their insurance premiums are not supporting total drug purchases and their purchases are not working to satisfy their deductible. Whether this would yield an overall increase in individual consumer cost would be dependent upon the premium differences between U.S. and foreign-sold drugs and the degree of cost-sharing outlined in that insurance plan.

Lastly, additional legal challenges from the pharmaceutical industry are likely once the first state-sponsored importation programs become an actuality. A potential challenge could stem from the addition of “states” as sponsors of demonstration importation programs in the Administration’s proposal. This is because Section 804 states “[t]he Secretary, after consultation with the United States Trade Representative and the Commissioner of Customs, shall promulgate regulations permitting pharmacists and wholesalers to import prescription drugs from Canada into the United States.” Thus yet another revision of the FDCA would seem necessary for the HHS to be able to legally authorize a state importation program and successfully defend the program under the scrutiny of the U.S. legal system.

## Conclusion

The authors believe drug importation alone is unlikely to be a panacea for rising pharmaceutical costs. Views on drug importation remain polarized with supporters and opponents seeking to justify their own interests. The durability of drug importation, under the limitations outlined in the FDCA, to lower drug costs is hampered by programs only being allowed to import pharmaceuticals from Canada and by excluding the most expensive therapies. However, the importation of a broader definition of pharmaceuticals from a broader array of industrial nations while codifying personal importation exemptions may offer U.S. consumers a degree of price relief for a moderate period of time while more sustainable strategies to reduce cost are pursued.

## Disclaimer

This manuscript has not been submitted or published elsewhere at the time of this submission.
